# The Threat of Weight-Loss Over the Counter Supplements: A Case of Camellia Sinensis Autoimmune Hepatitis

**DOI:** 10.7759/cureus.36023

**Published:** 2023-03-11

**Authors:** Daniel Hernan Sacoto, Valentina Turbay, Jagbir Sandhu, Shobhana Chaudhari, Juan Cosico

**Affiliations:** 1 Department of Internal Medicine, Metropolitan Medical Center/New York Medical College, New York, USA; 2 Department of Internal Medicine, Advocate Christ Medical Center, Chicago, USA; 3 Department of Pathology, Metropolitan Medical Center/New York Medical College, New York, USA

**Keywords:** liver function, herbal supplements, autoimmune hepatitis, green tea, elevated liver enzyme

## Abstract

Autoimmune hepatitis (AIH) arises as a result of environmental and immunological interactions. Herbal and dietary supplements (HDS) are known triggers, and approximately half of the U.S. adult population consumes them, even though they are restricted. Therefore, the importance of recognizing potential triggers of AIH is considered relevant. The mechanism behind HDS Camellia Sinensis inducing AIH is related to its compounds, catechins, which induce reactive oxygen species leading to a liver immune-mediated response. We present here a challenging case of a middle-aged woman with AIH following the consumption of a weight-loss Mexican green tea containing Camellia Sinensis.

## Introduction

Autoimmune hepatitis (AIH) is a chronic inflammatory liver disease where autoimmune-mediated elements against hepatocytes are thought to play a role [1]. This heterogeneous disease is considered a result of the interaction of multifactorial elements, including genetic and environmental triggers. Environmental insults are able to trigger an aberrant immunological reaction leading to autoimmune-mediated injury to hepatocytes in genetically susceptible individuals [2]. Hence, studies have suggested the importance of seeking and identifying environmental triggers in lifestyle, food, beverages, or chemicals that may trigger innate and adaptive immune responses leading to a breakdown of tolerance against hepatocytes [3]. Additionally, the importance of recognizing potential triggers of AIH is considered relevant since approximately half of the U.S. adult population consumes HDS, with a recent overall increase in its use [4,5]. This has been demonstrated by the increase in pocket costs, which were $14.8 billion in 2008 and increased to $30.2 billion in 2012 [6]. Therefore, it is paramount to raise awareness in the medical and non-medical communities about the risks of the indiscriminate use of HDS.

Middle-aged women have the highest risk of developing AIH [7]. The presentation may vary from asymptomatic to insidious. It can manifest with an acute onset of general fatigue or jaundice. Other cases may mimic acute viral hepatitis [8]. It is common to initially miss the diagnosis; in fact, making a diagnosis of AIH after progression to a cirrhotic stage is common in both developed and developing countries [9]. Therefore, it is extremely important to have a suspicion for AIH in the case of elevated transaminases, prompting a diagnosis at an early fibrotic stage [2].

The lack of particular markers for its diagnosis has made it a challenging disease [10]. A diagnostic tool has been created based on multiple validated criteria [11,12]. It commonly presents with laboratory abnormalities, including transaminitis, positive anti-nuclear antibodies (ANA) or anti-smooth muscle antibodies (SMA), or elevated immunoglobulin G (IgG) levels [2]. Ultimately, a liver biopsy is needed to establish the diagnosis. An interface hepatitis or plasma cell infiltration is considered a specific diagnostic pattern [13]. In our case, laboratory markers and histology were key determinants for the final diagnosis.

## Case presentation

A 42-year-old Hispanic female with type-2 diabetes mellitus presents to the emergency room complaining of a three-day history of mild (3/10 visual analog pain scale) stabbing right upper quadrant abdominal pain associated with nausea and non-bilious, non-bloody emesis. The patient has lived in the United States (U.S.) for the last 20 years. She denied recent travel. She doesn’t consume alcohol or use recreational drugs.

On arrival, she was normotensive (BP: 118/71 mmHg) and afebrile (Temp: 97.5 Fahrenheit). The physical exam was remarkable for icteric skin and sclerae, present bowel sounds, and a soft, non-distended abdomen without guarding or rebound but a positive Murphy sign.

Laboratory results were significant for total bilirubin: 5.3 mg/dL, direct bilirubin: 3.3 mg/dL, alkaline phosphatase: 222 U/L, aspartate aminotransferase (AST): 1160 U/L, alanine aminotransferase (ALT): 2092 U/L. R ratio was calculated to distinguish between a cholestatic, hepatocellular, or mixed injury pattern. The score was > 5, indicating a hepatocellular injury. Synthetic function (INR: 1.1 and albumin: 3.0 mg/dl) was preserved. Further laboratories showed no evidence of leukocytosis (6.29 K/uL). Lipase (23 U/L) and basic metabolic panel were normal.

**Figure 1:**
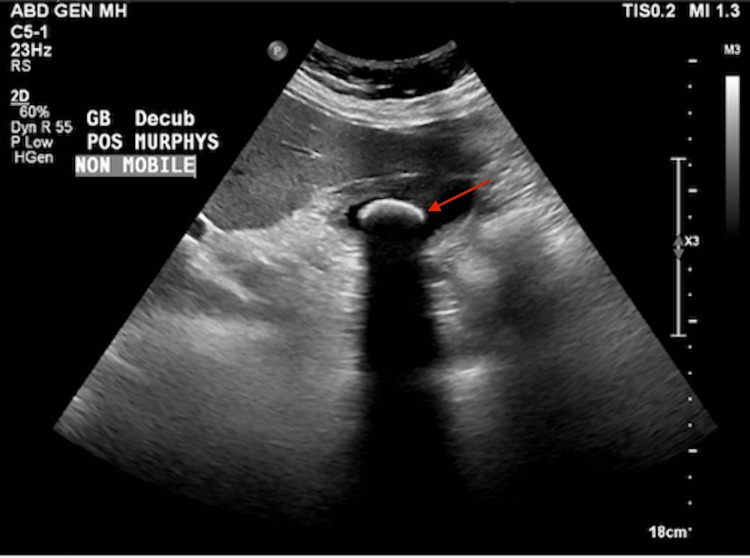
A liver ultrasound showed a non-mobile stone measuring up to 2.7 cm. No evidence of gallbladder wall thickening or pericholecystic fluid. CBD measures 5 mm.

**Figure 2:**
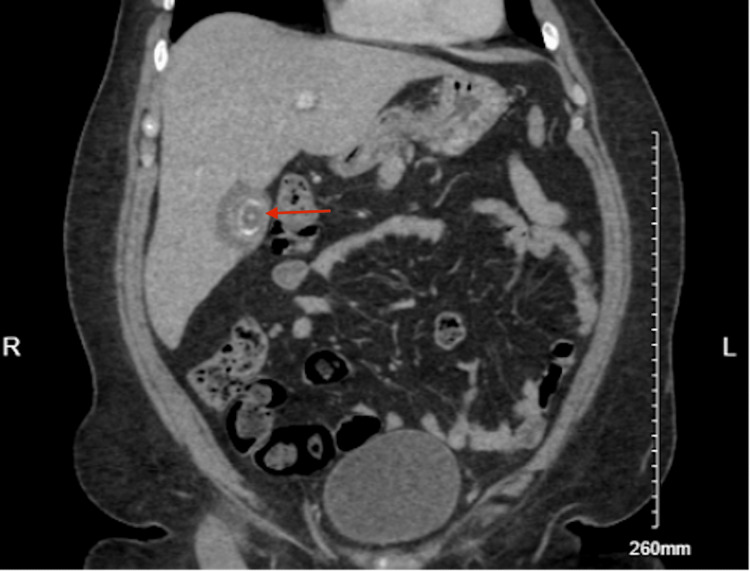
Abdomino-pelvic contrast CT-scan showed a mild degree of fluid around an under-distended gallbladder

**Figure 3:**
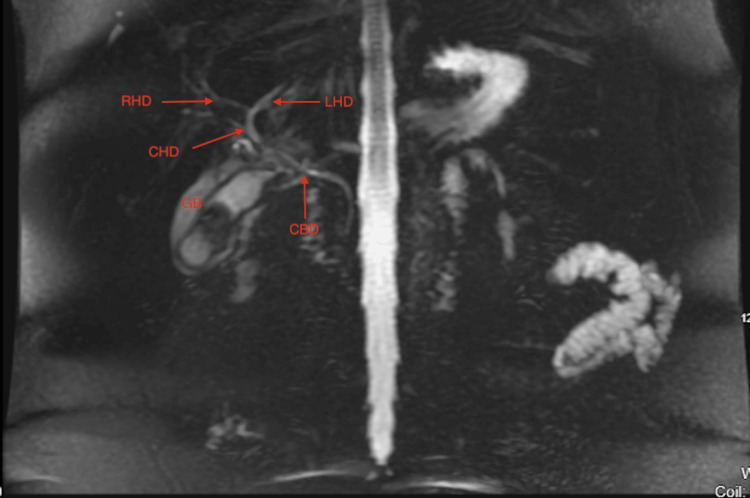
MRCP with no evidence of stones in the CBD. Underdistended gallbladder with stone and surrounding fluid RHD: Right hepatic duct, LHD: Left hepatic duct, CHD: Common hepatic duct, CBD: Common bile duct, GB: Gallbladder, MRCP: Magnetic resonance cholangiopancreatography

Abdominal ultrasound (US) showed a 2.7 cm non-mobile stone in the gallbladder; however, there was no evidence of wall thickening or pericholecystic fluid. At this point, based on the Tokyo guidelines 2018 (9), there was a suspicion of acute cholecystitis, which probably led to the liver injury. A computed tomography (CT) abdomen with contrast demonstrated an under-distended gallbladder with free fluid around, pointing towards a gallbladder perforation. A subsequent hepatobiliary iminodiacetic acid scan (HIDA scan) was performed, with no evidence of biliary perforation or obstruction. Magnetic resonance cholangiopancreatography (MRCP) was performed to better delineate the biliary anatomy. There was no evidence of stone, stricture on the (CBC) or cystic duct (Figures 1-3).

Based on the available evidence, liver injury secondary to biliary obstruction/cholecystitis was unlikely. Therefore, further assessment for hepatocellular injury was initiated. Ischemic hepatitis and Budd-Chiari syndrome were ruled out after a normal liver vasculature architecture was present on the liver Doppler US.

Workup for infectious causes was also conducted. There was no evidence of acute hepatitis A virus (HAV) (IgM non-reactive), acute hepatitis B virus (HBV) (HBsAg non-reactive; DNA polymerase chain reaction (PCR) non-detected), or hepatitis C virus (HCV) (antibody non-reactive). However, the Hepatitis E virus (HEV) was positive (IgM detected) with viral DNA <1800 IU/Ml). Further history revealed a recent sick contact, an eight-year-old daughter with a sore throat and fever. This prompted additional testing; Epstein-Barr Virus (EBV) IgM was non-detected, but cytomegalovirus (CMV) IgM was elevated (57.0 AU/mL) with a negative CMV PCR (no viral DNA detected). Other less common viral infections were considered: negative Herpes Simplex Virus type 1/2 (IgM non-detected), SARS-CoV-2 (PCR negative), and influenza A and B (PCR negative). Bacterial etiologies known to cause liver injury were also ruled out: leptospirosis (IgM non-detected), syphilis (Rapid Plasma Regain negative), and tuberculosis (Quantiferon negative).

As our patient was less than 45 years of age, Wilson's disease needed to be considered in addition to other metabolic conditions such as hemochromatosis and α-1-antitrypsin deficiency; ceruloplasmin (39 mg/dl) was within normal limits, iron levels (129 ug/dl) were within normal limits, and ferritin levels were mildly elevated (535 ng/mL), but it was considered an acute phase reactant due to the ongoing inflammatory process. Since, genetic disorders were considered as well: Alpha 1 Antitrypsin (179 mg/dl) levels were within normal limits. Autoimmune conditions were at the top of our differential diagnosis. Interestingly, anti-smooth muscle antibody (ASMA) (<1:20), mitochondrial antibody (AMA) (<1:20), and anti-LKM Ab (<20.1 Units) came back negative. However, antinuclear antibody (1:320) and IgG blood levels (2060 mg/dl) were elevated, setting the stage for AIH as a probable diagnosis.

Drug-induced liver disease (DILI) workup included acetaminophen levels, which were normal (<2.5). She denied the consumption of drugs associated with hepatocellular liver injury, including isoniazid, macrolides, nitrofurantoin, minocycline, anti-epileptics, and NSAIDs. Upon further questioning, the patient revealed intermittent consumption of green tea containing Camellia Sinensis for the last three months, a product brought from Mexico that she was using for weight loss.

**Figure 4:**
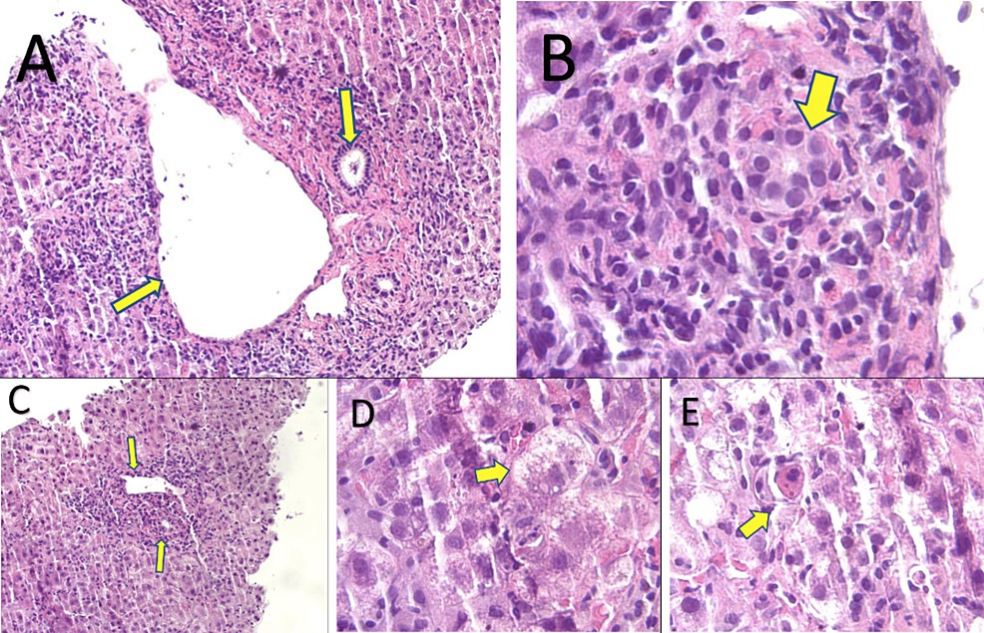
A) Portal lymphoplasmacytic infiltrate with interface hepatitis and bile duct injury, otherwise typical of autoimmune hepatitis. B) Magnified bile duct and presence of fibrosis. C) Lymphohistiocytic infiltrate in the sinusoids of the lobule, portal vein and bile duct. D) Hepatocyte ballooning E) Lymphohistiocytic infiltrate and an apoptotic hepatocyte is also found.

Hence, a diagnosis of drug-induced, or, in this case, herbal tea-induced, AIH was considered. A liver CT-guided biopsy demonstrated portal, periportal, and lobular inflammation with lymphocytic plasma cells and a few polymorphonuclear neutrophils associated with ballooning degeneration and apoptosis of hepatocytes (Figure 4).

The presence of interface hepatitis on histology, along with an AIH score of 22, was strongly suggestive of AIH. Type I AIH was most likely based on age, gender, a very elevated ANA, and a negative anti-LKM Ab. Even though primary biliary cholangitis was a diagnosis to consider, a negative AMA (<1:20) along with an incongruous histologic pattern (interlobular bile duct injury) did not support the diagnosis.

**Figure 5:**
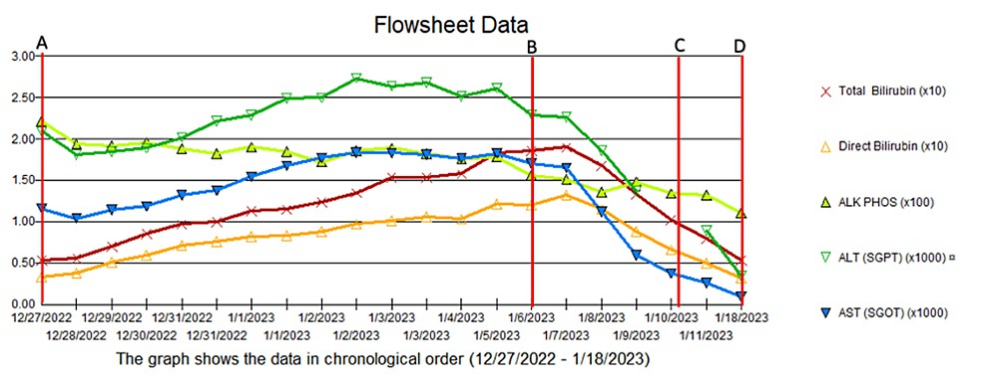
Liver function tests at different stages of the disease. A-B: from hospital admission to previous therapy initiation. B-C: the first seven days of therapy. C-D: the second 14 days of therapy.

During hospitalization, treatment with intravenous fluids and N-acetylcysteine did not resolve acute hepatitis, which further supported AIH. We followed the American Association for the Study of Liver Disease guidelines recommendations [33]. Prednisone monotherapy (60 mg/day) was started for seven days, followed by 40 mg/day for another week. At that time, the Thiopurine methyltransferase results came back normal. Then, from day 14 onward, we started a maintenance prednisone dose (30 mg/day) in addition to azathioprine (50 mg/day). The patient showed an overall positive response after the treatment (Figure 5).

Model for end-stage liver disease (MELD) Na score was calculated daily. In the pre-steroid treatment phase. the patient had a MELD-Na of 22, indicating a 19.5% estimated three-month mortality. After 14 days of treatment, the score decreased to six, showing a 6% mortality.

## Discussion

We report the case of a patient presenting with severe acute hepatitis in the setting of AIH after consumption of green tea (Camellia Sinensis) that improved after corticosteroid therapy.

The gradual inflammation in AIH is considered a result of an interplay between genetic predisposition, environmental triggers, and the unregulated response of the immune system [14]. Even though the majority of cases of AIH have an idiopathic origin, it is important to assess the consumption of herbal and dietary supplements (HDS) as they are implicated in approximately 18% of liver injury cases [15].

HDS is considered the most widely used complementary and alternative medicine, with an increasing trend over the last two decades [16]. Their use around the world is partially related to their perception of them as a safety measure to treat and prevent various diseases [17]. Its regulation is not dependent on the Food and Drug Administration (FDA); in fact, it does not require pre-marketing approval like conventional medications [18]. Nonetheless, the rising popularity of herbs and their association with liver injury is known and has prompted strategies to increase public knowledge regarding its complications. Currently, there are internet databases such as LiverTox [19] and Hepatox [20], which provide a list of hepatotoxic drug names available to the public.

The diagnosis of HDS-induced liver injury is challenging because there are no specific markers, and most of them are not FDA-approved, so their side effects are underreported [17]. Therefore, high suspicion is needed since the presentation can vary from transient elevation of liver function tests to fulminant hepatic failure resulting in death or liver transplantation [21]. AIH can present with any pattern of liver injury, including hepatotoxic, cholestatic, or mixed [22]. Based on the R ratio [23], we believed that our patient suffered a hepatotoxic predominant injury. When characterizing the type of HDS compound, it is important to determine if it contains androgenic anabolic steroids (AAS) or non-anabolic steroids, the latter producing a more severe presentation with higher morbidity and mortality [24]. Our patient's HDS contained non-anabolic steroids, which explained the significantly elevated transaminases at presentation.

HDS can also present as DILI as a result of direct liver injury. A systematic review and meta-analysis identified 79 different types of herbs and herbal compounds associated with the development of liver injury. Among them, He-Shou-Wu, Herbalife, kava kava, Greater celandine, germander, Hydroxycut, skullcap, kratom, Gynura segetum, garcinia Cambogia, ma huang, chaparral, senna, aloe vera, and Green tea extract were the most commonly identified [25]. HDS can also elicit indirect liver damage after inducing an immune-mediated response which leads to AH. A literature review identified 8 case reports of AIH induced by the Chinese herb Xiang-tian-Guo which contains Swietenia macrophylla seeds, frequently used to treat diabetes and hypertension [26].

The Chinese green tea (Camellia Sinensis) consumed by our patient has been used for centuries, and it was considered safe among the public. However, liver injury may develop within three months of its use [27]. Several cases have been reported of DILI caused by Camellia Sinensis [28-33]. We identified only one case of autoimmune liver hepatitis whose onset was triggered by the consumption of green tea infusion in a patient previously taking oral contraceptives and irbesartan [27].

The mechanism of AIH triggered by Camellia Sinensis is not well elucidated. It is thought that one of its compounds, Catechins such as epigallocatechin-3-gallate (EGCG), are likely to be involved [27]. EGCG induces reactive oxygen species, which leads to an idiosyncratic or immune-mediated response [27]. Gallo et al. further explained this response as a result of the patient's genetic susceptibility [34]. EASL guidelines support the diagnosis of drug-induced AIH when the allele DRB115:01 is present [10]. Unfortunately, this test was not available in our hospital. Carriers of these genetic variants of hepatic metabolisms can become susceptible to the oxidative stress of HDS. The altered hepatic metabolism can further increase the herb concentration, favoring the haptenization of liver proteins, eventually leading to the development of AIH [35]. This theory is called the “hapten hypothesis”, where haptens or proteins may be recognized by the immune system as neoantigens and trigger an immunocyte activation that generates autoantibodies and cell-mediated immune responses [35, 36]. In the context of a female, <45 years old patient, probably genetically susceptible, we suggest that the EGCG could have triggered an immune-mediated process that led to AIH.

Herbal compounds used for weight loss preparation are mostly combined with other chemicals, which may also trigger an immune response [37]. Additionally, the concern about the wide use of pesticides while growing green tea trees has been previously discussed [38]. This led us to think about potential pesticide-induced AIH, especially from less regulated products ordered from developing countries.

Guidelines regarding initiation of therapy vary; however, those with advanced or active AIH (advanced fibrosis, cirrhosis, ALT ≥3 times the upper limit of normal) are an absolute indication for treatment [39]. In our case, even though there was no evidence of cirrhosis, the extremely elevated transaminases granted initiation of therapy. Patient follow-up demonstrated biochemical remission, which is the aim of AIH treatment.

## Conclusions

HDS-triggered AIH is underreported. Therefore, it is essential to create a culture of knowledge and trust among patients, encouraging the disclosure of HDS use through a non-judgmental approach. A thorough clinical history, including HDS consumption, is key to identifying AIH triggers. Certain patients carry genetic variants that alter their hepatic metabolism, making them susceptible to oxidative stress produced by toxic insults such as HDS.
